# Multi-Scale Global Contrast CNN for Salient Object Detection

**DOI:** 10.3390/s20092656

**Published:** 2020-05-06

**Authors:** Weijia Feng, Xiaohui Li, Guangshuai Gao, Xingyue Chen, Qingjie Liu

**Affiliations:** 1 College of Computer and Information Engineering, Tianjin Normal University, Tianjin 300387, China; 2 Postdoctoral Innovation Practice Base, Huafa Industrial Share Co., Ltd., Zhuhai 519000, China; 3 Key Wireless Laboratory of Jiangsu Province, School of Telecommunication and Information Engineering, Nanjing University of Posts and Telecommunications, Nanjing 210003, China; 2016010102@njupt.edi.cn; 4 School of Computer Science and Engineering, Beihang University, Beijing 100191, China; gaoguangshuai1990@buaa.edu.cn (G.G.); buaachen1993@foxmail.com (X.C.); 5 Hangzhou Innovation Institute, Beihang University, Hangzhou 310051, China; qingjie.liu@buaa.edu.cn

**Keywords:** visual saliency, multi-scale, global contrast, CNN

## Abstract

Salient object detection (SOD) is a fundamental task in computer vision, which attempts to mimic human visual systems that rapidly respond to visual stimuli and locate visually salient objects in various scenes. Perceptual studies have revealed that visual contrast is the most important factor in bottom-up visual attention process. Many of the proposed models predict saliency maps based on the computation of visual contrast between salient regions and backgrounds. In this paper, we design an end-to-end multi-scale global contrast convolutional neural network (CNN) that explicitly learns hierarchical contrast information among global and local features of an image to infer its salient object regions. In contrast to many previous CNN based saliency methods that apply super-pixel segmentation to obtain homogeneous regions and then extract their CNN features before producing saliency maps region-wise, our network is pre-processing free without any additional stages, yet it predicts accurate pixel-wise saliency maps. Extensive experiments demonstrate that the proposed network generates high quality saliency maps that are comparable or even superior to those of state-of-the-art salient object detection architectures.

## 1. Introduction

Salient object detection (SOD) is a fundamental task in computer vision that attempts to mimic human visual systems that rapidly respond to visual stimuli and locate visually salient objects in a scene. Estimating salient regions from an image could facilitate a lot of vision tasks, ranging from low-level ones such as segmentation [1] and image resizing [2] to high-level ones such as image captioning [3]; thus, it has been receiving increasing interest in the computer vision community and has been extended to other relevant topics, such as video SOD [4,5] and RGB-D SOD [6,7]. Numerous methods have been developed in the past decades. Most of them focus on two topics; the first one works on predicting eye fixations, and the other one aims at detecting salient object/regions from an image. In this work, we mainly focus on the latter one, i.e., detecting salient objects from clutter scenes.

Since the pioneer work of Itti’s computational saliency model [8], extensive efforts have been devoted to develop saliency methods identifying objects or locating regions that attract the attention of a human observer at the first sight of an image. Most of these methods draw inspiration from bottom-up human visual attention mechanisms, e.g., Feature Integration Theory (FIT) [9], and dedicate to measure uniqueness, distinctness and rarity of scenes to infer saliency maps, where the basic assumption is that objects might be salient if they differ from their surroundings. The central idea of these models is capturing contrast information of visual features either in a local manner [10] or in a global manner [11,12].

Since saliency computation is simulation of low-level stimuli-driven attention, early approaches mainly rely on low level features such as color, intensity, texture, etc. However, methods employing only low level features are very hard to produce satisfactory results. They suffer from the limitation that the detected regions may contain only parts of the salient objects, and the background is easily mixed up with the salient regions. One solution to overcome these limitations is extracting features from small regions (such as super-pixels) instead of pixels to incorporate context information [12]; another one is combining mid- or high-level cues [13] to introduce semantic knowledge of the salient objects. These methods could obtain promising results; however, they may fail to detect salient objects embedded in clutter backgrounds or sharing similar appearance with some distractors. Salient object detection remains a challenging problem especially under such complex scenes.

Recently, deep convolutional neural networks (CNNs) have achieved astonishing performance on a variety of computer vision tasks, such as object detection [14] and image segmentation [15]. The great success of CNNs has been motivating researchers to employ CNNs as feature extractors to represent salient objects [16,17]. In these methods, saliency can be estimated in pixel level or in region level. Inspired by FCN [15], some works model saliency computation as a dense labeling problem, they directly learn mapping functions from input images to their saliency maps [18]. These methods, which impel CNNs to learn internal features that capture saliency information of images in an implicit way, are pure computational methods, and they do not explain the psychophysical evidence. Given the fact that visual contrast is the most important factor in visual attention, capturing contrast information is the key idea in designing biologically-inspired saliency models. Therefore, many CNN based methods employ contrast strategy to build network architectures, in which a super-pixel segmentation step is usually added to obtain homogeneous regions, and contrast measurement between them is computed by using CNN features [16,17].

**Figure 1:**
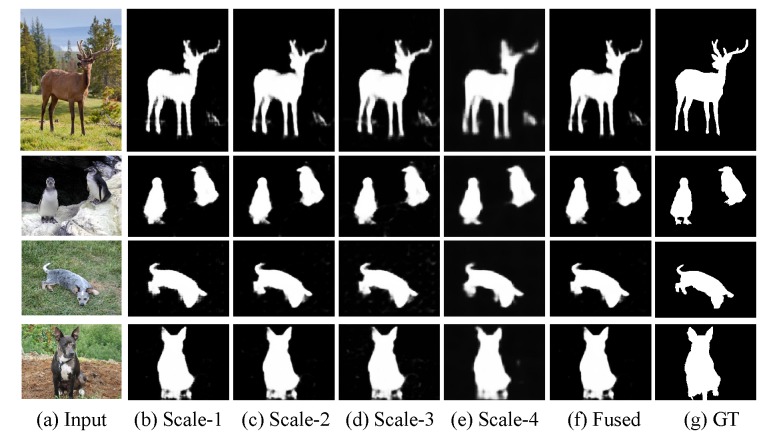
Saliency maps generated by the proposed multi-scale global contrast convolutional neural network (CNN). Our method predicts saliency maps in multiple scales and fuses them to obtain the final results.

Recall that global (contrast) information is critical when detecting salient objects [12], and neurons in high level layers of a CNN have large receptive fields that could cover the entire image, thus capturing its global information, while neurons in low level layers only possess small receptive fields capturing local information. Given local and global features of an image, we can design a CNN architecture that learns their contrast information, thus estimating a saliency map of the image. Based on this idea, in this paper, we develop an end-to-end CNN architecture to automatically capture global contrast information between each local feature and the global image feature. Furthermore, we implement it in a multi-scale manner to leverage different level features; see Figure 1 for an example. We test the proposed method on four widely used saliency datasets and compare it with 10 state-of-the-art models. Experimental results demonstrate that the proposed method could effectively detect salient objects and achieve comparable or even superior performance to that of the state-of-the-art ones.

The rest of this paper is organized as follows. In Section 2, we give a brief review on contrast and CNN based saliency methods. Section 3 elaborates the detail architecture of the proposed multi-scale global contrast CNN. Section 4 gives experiments and comparisons with the state-of-the-art methods. Finally, this paper is concluded in Section 5.

## 2. Related Work

A great number of salient object detection methods have been proposed in the past decades; a comprehensive survey can be found from [19]. In this section we give a brief review of saliency computation models closely related to our method.

### 2.1. Contrast Based Models

Recent studies [20] have suggested that visual contrast is at the central of saliency attention. Most existing visual saliency computation models are designed based on either local or global contrast cues.

Local contrast based methods investigate the rarity of image regions with respect to their local neighborhoods [12]. The pioneer work on these models is Itti’s model [8], in which saliency maps are generated by measuring center-surround difference of color, orientation, and intensity features. Later, Harel et al. [21] estimates center-surround saliency maps in a graph computation manner and achieves superior performance to that of Itti’s model. Similarly, Klein et al. [22] encodes local center-surround divergence in multi-feature channels and computes them in an efficient scale-space to deal with scale variations. Liu et al. [23] incorporates multi-scale contrast features with center-surround histogram and color spatial distribution by Markov random fields to detect salient objects. Without knowing the size of the salient object, contrast is usually computed at multiple scales. Jiang et al. [24] integrates regional contrast, regional property and regional background descriptors to form saliency maps. One major drawback of local contrast based methods is that they tend to highlight strong edges of salient objects thus producing salient regions with holes.

Global contrast based methods compute saliency of a small region by measuring its contrast with respect to all other parts of the image. Achanta et al. [11] proposes a simple frequency-tuned salient region detection method, in which saliency value of a pixel is defined as difference between its color and mean color of the image. Cheng et al. [12] introduces a global contrast based salient object detection algorithm, in which saliency of a region is assigned by the histogram difference between the target region and all other regions. Later, they propose a soft image abstraction method to capture large scale perceptually homogeneous elements, which enables more effective estimation of global saliency cues [25]. Differently, in [10], contrast and saliency estimation is formulated in a unified way using high-dimensional Gaussian filter.

### 2.2. Cnn Based Models

Representing pixels or regions efficiently and compactly is critical for saliency models. The aforementioned methods only employ low-level features such as color and texture. Recently, inspired by the great success of CNNs in many computer vision tasks, researchers in the community are encouraged to leverage power of CNNs to capture high level information from the images. Vig et al. [26] is probably the first attempt at modeling saliency computation using deep neural networks. This work focuses on predicting eye fixation by assembling different layers using a linear SVM. Zhao et al. [16] and Li et al. [27] extract a global feature of an image and a local feature of a small region in it using different CNNs, and then, the saliency of this region is formulated as a classification problem. Wang et al. [28] proposes a saliency detection model composed of two CNNs; one learns features capturing local cues such as local contrast, textures and shape information, and the other one learns the complex dependencies among global cues. He et al. [29] learns the hierarchical contrast features using multi-stream CNNs. To obtain accurate salient boundaries, images are first segmented into super-pixels in multi-scales. Two sequences, color uniqueness and color distribution, are extracted from each super-pixel and fed into CNNs to obtain features. Saliency maps are generated by fusing saliency results inferred from each scale. Li et al. [17] adopts a two-stream deep contrast CNN architecture. One stream accepts original images as input, infers semantic properties of salient objects and captures visual contrast among multi-scale feature maps to output their coarse saliency maps. The other stream extracts segment wise features and models visual contrast between regions and saliency discontinuities along region boundaries. Ren et al. [30] puts forward a multi-scale encoder-decoder network (MSED) by fusing multi-scale features from the image-level. Li et al. [31] presents a multi-scale cascade network (MSC-Net) for saliency detection in a coarse-to-fine manner, which encodes abundant contextual information whilst progressively incorporating the saliency prior knowledge to improve the detection accuracy. Li et al. [32] discloses the importance of inference module in the saliency detection and presents a deep yet lightweight architecture which extracts multi-scale features by leveraging a multi-dilated depth-wise convolution operation. Different from them, in this paper, we design an end-to-end multi-scale global contrast network that explicitly learns hierarchical contrast information among global and local features of an image to infer its salient object regions. Compared with the aforementioned multi-scale CNN-based models, our proposed model is lightweight and without any pre-processing operations.

## 3. Multi-Scale Global Contrast CNN

In this section, we will give details of our multi-scale global contrast CNN (denoted as MGCC) architecture.

### 3.1. Formulation

Salient object detection can be considered as a binary classification problem. Given an image *I*, the saliency value of a pixel *i* (*i* also could be a super-pixel) in it can be represented as follows,

(1) $$S_{i}^{I}=\mathrm{Pr}\left(y_{i}=1|f_{i},f_{I};W\right)$$

where $S^{I}$ is the saliency map of the image *I* (for notational simplicity, we will drop the superscript *I* in the remainder of this paper), $S_{i}$ is the saliency value of pixel *i*, $f_{i}$ and $f_{I}$ are features of the pixel *i* and image *I*, respectively. $y_{i}=1$ indicates the probability of the pixel *i* being salient, while $y_{i}=0$ indicates background, W is collection of parameters.

In global contrast based methods, $S_{i}$ can be estimated through measuring the distance of the two features,

(2) $$S_{i}=C\left[d(f_{i},f_{I});W\right]$$

where C[·] is a function estimating saliency maps from d(·), and d(·) is a metric function measuring the distance of $f_{i}$ and $f_{I}$, which could be a simple Euclidean distance or other pre-defined distance metrics. For example, in [12], features are represented using color histograms, the saliency of a super-pixel is defined as its color contrast to all other regions in the image, which can be inferred from the weighted summation of color distances between the current region and all other ones.

Since $S_{i}$ is a probability value ranging from 0 to 1, C[·] often adopts the following form,

(3) $$S_{i}=C\left[d(f_{i},f_{I});W\right]\propto \sigma \left(d(f_{i},f_{I});W\right)$$

where σ(·) is a nonlinear function, e.g., sigmoid function, mapping d(·) to [0,1]. If we represent $f_{i}$ and $f_{I}$ using deep features and define d(·) as a metric learned from training data, then Equation (3) can be solved using a convolutional neural network. In the following section, we will give details of the proposed network architecture to achieve this.

### 3.2. Global Contrast Learning

The essence of obtaining contrast information between two regions is quantifying a “distance" between features of them, thus inducing a measure of similarity. As discussed above, the function d(·) can be viewed as a metric function that captures distance between $f_{i}$ and $f_{I}$, in which larger distance indicates higher contrast, thus higher probability being salient. There are multiple ways to calculate d(·). For instance, it can be formulated as pre-defined metrics, such as $L_{1}$ or $L_{2}$ norms. However, this requires the two features to have the same dimension, which is hard to achieve in CNNs. Suppose $f_{i}^{l}$ is a feature of pixel *i* extracted from the *l*-th convolutional layer of a CNN (e.g., VGG-16 [33]). Although we can apply global pooling on this layer to obtain $f_{I}$, thus making these two features have the same dimension, i.e., the channels of feature maps in this layer, lots of information will be lost during pooling process, especially when *l* is in low layers. Furthermore, low level features lack of semantic information, which is very important in detecting salient objects [34]. An alternative solution is adding an additional layer to project both of them into an embedding space, making them to have equal size and then calculating a distance matrix. However, it is hard to achieve satisfactory results by inferring salient objects directly from distance matrices; this is mainly because important semantic information about the salient objects is missing when computing distances.

**Figure 2:**
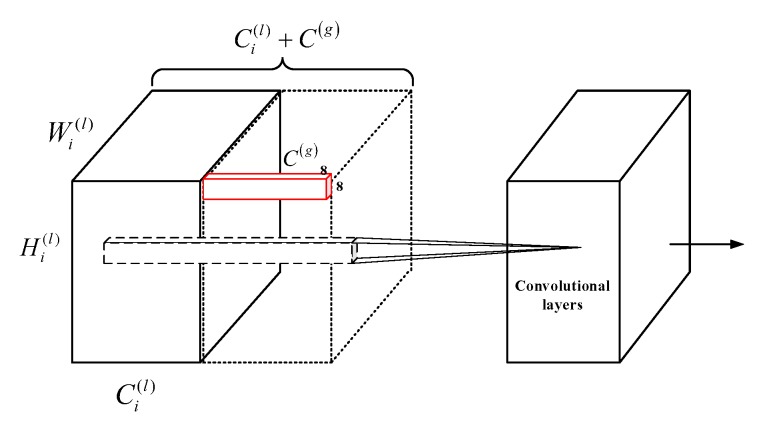
A sketch of learning global contrast module used in this paper. $W_{i}^{\left(l\right)}$, $H_{i}^{\left(l\right)}$ and $C_{i}^{\left(l\right)}$ respectively represent the width, height and channel of the feature maps at the previous layers, and $C^{\left(g\right)}$ means the channel of the global feature map, resizing the global feature map to the same size as the feature maps at previous layers and concatenating them in a channel-wise manner.

In addition to the pre-defined metrics, another solution is defining metric with the knowledge of the data, that is, learning the metric functions from the training samples. As a powerful tool as it is, CNNs have been proved to be very effective in approximating very complex functions and in learning visual similarities. To achieve this end, we attempt to design a CNN architecture that learns the distance function between $f_{i}$ and $f_{I}$. One important thing that should be noted is that the semantic information of the object should be preserved because we intend to recover accurate object boundaries. To achieve this, we design a very simple architecture that could capture global contrast of $f_{i}$ and $f_{I}$. Firstly, VGG-16 [33] is employed to extract features from input images. VGG-16 consists of 13 convolutional layers, 5 pooling layers and 3 fully connected layers. We modify it by removing the last 3 fully connected layers and using 256×256 input instead of original 224×224. The last pool layer of the modified VGG-16 (with size of 8×8×512) is used to represent the global feature. To emphasize contrast information and reduce distractions from semantic information, we apply an additional 1×1 convolutional layer to obtain compact 8×8×256 representations of global features. Then, we concatenate it with previous layers in a recurrent manner, and introduce more convolutional layers to learn visual contrast information, as shown in Figure 2. At the end of the network, output is up-sampled to meet the size of the ground truth maps. Although it is simple, this repeating concatenation strategy can successfully characterize contrast information of the image while preserving semantic information of salient objects.

### 3.3. Multi-Scale Global Contrast Network

Layers in a CNN from low to high levels capture different levels of abstraction. Neurons in early layers have small receptive fields that only respond to local regions of an input, thus producing low level features representing texture, edges, etc., while neurons in late layers have large receptive fields that may cover most of or even the entire image, thus capturing semantic information of the image or objects in it.

**Figure 3:**
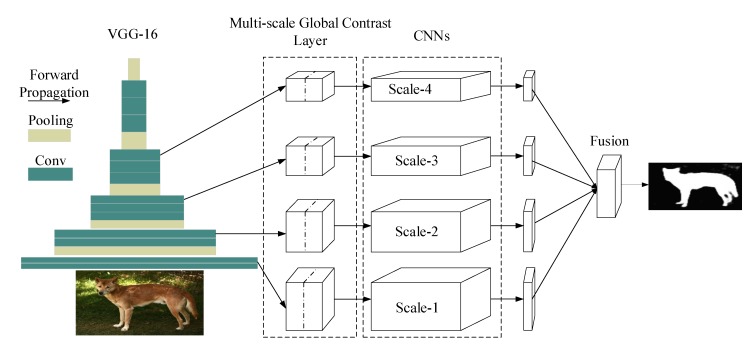
The architecture of the proposed multi-scale global contrast CNN (MGCC).

It is very important to employ low level features when generating output with accurate and clear boundaries [15]. Inspired by HED [35], we design multi-scale outputs to capture features in different layers and integrate them together to produce finer results. Specifically, we propose a Multi-scale Global Contrast CNN, abbreviated as MGCC, which adopts truncated VGG16 as the backbone, there are five convolutional segments, each of which contain two or three convolution layers, followed by one pooling layer to down-sample the size of the feature maps. Our proposed model takes the final output feature map, i.e., the fifth convolution segment, as the global feature. Then, we concatenate it with previous layers in a recurrent channel-concatenation manner by first resizing the global feature map to the same size with the corresponding feature maps at previous layers (the global contrast module, which corresponds to the left-part in Figure 2). This process is somewhat similar to feature pyramid network (FPN) [36] but different from it in that we respectively take the outputs of the previous four layers to concatenate with the fifth convolution layer, i.e., global features. For example, the output feature map of the fourth segment has the size of 16×16×256; thus, we resize the global feature whose size is 8×8×512 by upsampling two times, to the size of 16×16×512. Then, we concatenate them in a channel-wise manner. To learn more visual contrast information, we introduce several more convolutional layers (referred to the right-part of Figure 2). Consequently, the proposed MGCC generates four scale outputs, each of which could produce accurate saliency maps. We resize all the saliency maps of the four scale outputs to the same size of the original image and then fuse them in an element-wise summation to obtain the final finer saliency map. Figure 1 shows several examples. The architecture of the proposed MGCC is shown in Figure 3. The detail parameters are given in Table 1.

As discussed above, the salient object detection task can be formulated as a binary prediction problem; thus we use binary cross entropy as loss function to train our network. Given a set of training samples ${\left\{(X^{n},Y^{n})\right\}}_{n=1}^{N}$, where *N* is the number of samples, $X^{n}$ is an image, and $Y^{n}$ is the corresponding ground truth, the loss function $L^{m}$ for the *m*-th scale output is defined as

(4) $$\begin{array}{cc} L^{m}\left(W^{m},W^{\mathrm{vgg}}\right) & =-\sum_{j=1}^{\left|Y\right|}Y_{j}\log\mathrm{Pr}\left({\hat{Y}}_{j}^{m}=1|X;W^{m},W^{\mathrm{vgg}}\right)\\ \; & +\left(1-Y_{j}\right)\log\mathrm{Pr}\left({\hat{Y}}_{j}^{m}=0|X;W^{m},W^{\mathrm{vgg}}\right) \end{array}$$

where ${\hat{Y}}_{j}^{m}$ is the predicted saliency value for pixel *j*. The fused loss $L^{\mathrm{fused}}$ takes a similar form to Equation (4), and the fusion weights w are also learned form training samples. Finally, the loss function for training is given by

(5) $$L\left(W\right)=\alpha L^{\mathrm{fused}}\left(W\right)+\sum_{m=1}^{4}\beta^{m}L^{m}\left(W^{m},W^{\mathrm{vgg}}\right)$$

where $W=\{W^{\mathrm{vgg}},W^{1},\ldots ,W^{4},w\}$ is the collection of the parameters in the proposed network. w denotes the trainable parameters in the additional convolution layer for scale-4, which has been described in Table 1. α and βs are weights balancing different loss functions and all set to 1 in our experiments.

## 4. Experiments

### 4.1. Datasets

All experiments are conducted on four widely used datasets, including ECSSD [37], HKU-IS [27], PASCAL-S [38] and DUT-OMRON [39].

- ECSSD [37] is a challenge dataset which contains 1000 images with semantically meaningful but structurally complex natural contents.
- HKU-IS [27] is composed by 4447 complex images, each of which contains many disconnected objects with diverse spatial distribution. Furthermore, it is very challenging for the similar foreground/background appearance.
- PASCAL-S [38] contains a total of 850 images, with eye-fixation records, roughly pixel-wise and non-binary salient object annotations included.
- DUT-OMRON [39] consists of 5168 images with diverse variations and complex background, each of which has pix-level ground truth annotations.

### 4.2. Evaluation Metrics

Three metrics, including precision-recall (P-R) curves, **F**-measure and Mean Absolute Error (MAE) are used to evaluate the performance of the proposed and other methods. For an estimated saliency map with values ranging from 0 to 1, its precision and recall can be obtained by comparing the thresholded binary mask with the ground truth. Making these comparisons at each threshold and averaging them on all images will generate P-R curves of this dataset.

The **F**-measure is a harmonic mean of average precision and recall, which is defined as,

(6) $$F_{\beta }=\frac{(1+\beta^{2})\cdot \mathrm{precision}\cdot \mathrm{recall}}{\beta^{2}\cdot \mathrm{prcision}+\mathrm{recall}}$$

As suggested by many existing works [16,40], $\beta^{2}$ is set as 0.3. MAE reflects absolute difference of the estimated *S* and the ground truth saliency maps *G*.

(7) $$\mathrm{MAE}=\frac{1}{W\times H}\sum_{x=1}^{W}\sum_{y=1}^{H}\left|S\left(x,y\right)-G\left(x,y\right)\right|$$

where *W* and *H* are width and height of the maps.

Both metrics of MAE and F-measure are based on pixel-wise errors and often ignore the structural similarities, as demonstrated in [41,42]. In many applications, it is desired that the results of the salient object detection model retain the structure of objects. Therefore, three more metrics, i.e., weighted F-measure $F_{\beta }^{w}$ [41], S-measure ($S_{\alpha }$) [42] and E-measure ($E_{m}$) [43] are also introduced to further evaluate our proposed method.

Specifically, $F_{\beta }^{w}$ [41] is computed as follows:

(8) $$F_{\beta }^{w}=\frac{\left(1+\beta^{2}\right)\cdot \mathrm{precisio}n^{w}\cdot \mathrm{recal}l^{w}}{\beta^{2}\cdot \mathrm{prcisio}n^{w}+\mathrm{recal}l^{w}}$$

where $\mathrm{precisio}n^{w}$ and $\mathrm{recal}l^{w}$ are the weighted precision and recall. Note that the difference between $F_{\beta }^{w}$ and $F_{\beta }$ is that it can compare a non-binary map against ground-truth with thresholding operation, to avoid the interpolation flaw. As suggested in [41,44,45,46], we empirically set $\beta^{2}=0.3$.

$S_{\alpha }$ [42] is proposed to measure the spatial structure similarities between saliency maps.

(9) $$S_{\alpha }=\alpha \times S_{o}+\left(1-\alpha \right)\times S_{r}$$

where α is a balance parameter between object-aware structural similarity $S_{o}$ and region-aware structural similarity $S_{r}$, as suggested in [42,47,48].

E-measure ($E_{m}$) [43,44,49,50] is to evaluate the foreground map (FM) and noise, which can correctly rank the maps consistent with the application rank.

(10) $$E_{m}=\frac{1}{w\times h}\sum_{x=1}^{w}\sum_{y=1}^{h}\phi_{FM}\left(x,y\right)$$

where ϕ denotes the enhanced alignment matrix, which is to capture pixel-level matching and image-level statistics of a binary map.

### 4.3. Implementation Details

We implement the proposed network in PyTorch [51]. As mentioned above, we utilize VGG-16 [33] pre-trained on ImageNet [52] as backbone to extract features. The MSRA10K dataset [12] is employed to train the network. Before feeding into the network, all images are resized to 256×256. During training, parameters are optimized using Adam optimizer. The learning rates for VGG-16 and other newly added layers are initially set as $10^{-4}$ and $10^{-3}$ and decreased by a factor of 0.1 in every 30 epochs. In addition, we set momentum as 0.9. The training was conducted on a single NVIDIA Titan X GPU with a batch size of 8. It will converge in 80 epochs. It should be noted that no data augmentation was used during training.

### 4.4. Comparison with the Sate-of-the-Art

We compare the proposed MGCC with 10 state-of-the-art saliency models, including 5 CNN based methods: LEGS [28], MDF [27], MCDL [16], ELD [53], DCL [17] and 5 classical models: SMD [40], DRFI [24], RBD [54], MST [55] and MB+ [56]. These methods are chosen because the first 5 are also CNN and contrast based methods, and the last five traditional methods are either reported as benchmarking methods in [19] or developed recently. For fair comparison, we employ either implementation or saliency maps provided by the authors.

**Figure 4:**
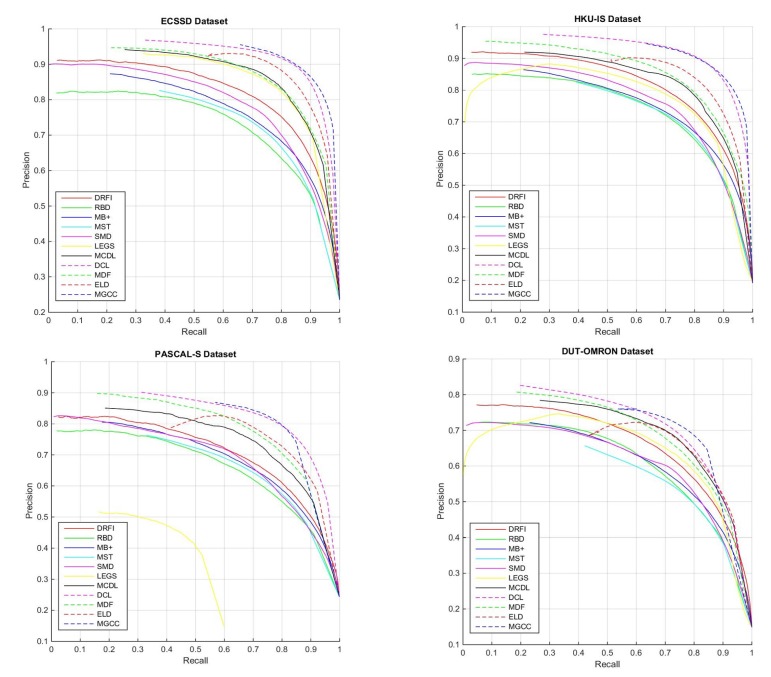
P-R curves of the proposed and 10 state-of-the-art methods on ECSSD, HKU-IS, PASCAL-S and DUT-OMRON datasets.

We report P-R curves in Figure 4 and list Max **F**-measure (Max$F_{\beta }$), MAE, $F_{\beta }^{w}$, $S_{\alpha }$ and $E_{m}$ in Table 2. From Figure 4 we can see that our method achieves better P-R curves on the four datasets; especially on ECSSD and HKU-IS datasets, it obtains the best results, showing that MGCC can achieve the highest precision and the highest recall comparing with other methods. On PASCAL-S and DUT-OMRON datasets, although MGCC drops faster than DCL [17] and ELD [53] on the right side of the P-R curves, we can observe that the MGCC obtains better or at least comparable break-even points (i.e., the points on the curves where precision equals recall), which indicates that our method can keep a good balance between precision and recall.

From Table 2, we can see that deep learning based approaches significantly outperform traditional saliency models, which clearly demonstrate the superiority of deep learning techniques. Among all the methods, the proposed MGCC achieves almost the best results over all the four datasets, except for the HKU-IS dataset, on which, DCL, a leading contrast based saliency model, performs slightly better than ours in terms of Max$F_{\beta }$ and $F_{\beta }^{w}$; however, it underperforms ours in terms of MAE, $S_{\alpha }$, and $E_{m}$. The proposed MGCC and DCL [17] obtain identical Max$F_{\beta }$ on the PASCAL-S dataset, yet lower MAE is achieved by our MGCC. It can be seen that MGCC improves MAE with a considerable margin on all four datasets. This demonstrates that our method can produce more accurate salient regions than other methods.

**Figure 5:**
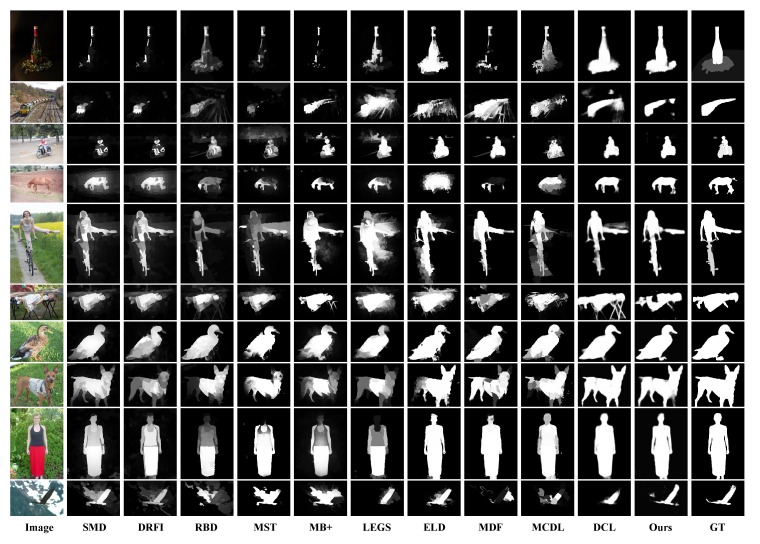
Visual comparison with the state-of-the-art methods.

Some example results of our and other methods are shown in Figure 5 for visual comparison, from which we can see our method performs well even under complex scenes. It is worth mentioning that to achieve better performance and obtain accurate salient regions, many CNN based models adopt two- or multi-stream architectures to incorporate both pixel-level and segment-level saliency information [16,17,27,28,53]. For instance, DCL consists of two complementary components; one stream generates low resolution pixel-level saliency maps, and one stream generates full resolution segment-level saliency maps. They combine the two saliency maps to obtain better results. While our network only has one stream and predicts saliency maps in pixel wise, with simpler architecture and without additional processing (e.g., super-pixel segmentation or CRF), our method achieves comparable or even better results than other deep saliency models. Another thing that should be mentioned is that, with simple architecture and completely end-to-end feed-forward inference, our network produces saliency maps at a near real time speed of 19 fps on a Titan X GPU.

### 4.5. Ablation Study

To further demonstrate the effectiveness of the multi-scale fusion strategy, we compare our proposed model with the results output from scale-1, scale-2, scale-3, and scale-4, as illustrated in Table 2.

From Table 2, we can observe that when merging global feature with the features of previous layers, the performance gradually increases from scale-1 to scale-4, which verifies that merging higher-level semantic features can further boost the performance. Additionally, from the metrics, we can see that fusing multi-scale information (i.e., our proposed MGCC model), the performance has significantly improved, which indeed demonstrates the effectiveness and superiority of our proposed multi-scale fusion strategy.

## 5. Conclusions and Future Work

In this paper, we have proposed an end-to-end multi-scale global contrast CNN for salient object detection. In contrast to previous CNN based methods, designing complex two- or multi-stream architectures to capture visual contrast information or directly mapping images to their saliency maps and learning internal contrast information in an implicit way, our network is simple yet good at capturing global visual contrast, thus achieving superior performance both at detecting salient regions and processing speed.

As demonstrated in existing literature [57], the SOC dataset [58] is the most challenging dataset. Some attempts have been made on this dataset in Deepside [44] and SCRNet [59]. We look forward to conducting some experiments on this dataset in our future work to further demonstrate the effectiveness and superiority of our proposed approach.

## Figures and Tables

**Table 1:** Detail architectures of the proposed network. (m,n)/(k×k) means that there are *m* channels in previous layer and *n* channels in current layer; the filters connecting them have size k×k. Scale-4 architecture is slightly different to the other three ones in that it has one additional convolutional layer.

	Layer 1	Layer 2	Layer 3	Layer 4	Layer 5
Scale-1	(320, 320)/(3×3)	(320, 256)/(1×1)	(256, 256)/(3×3)	(256, 1)/(1×1)	-
Scale-2	(384, 384)/(3×3)	(384, 256)/(1×1)	(256, 256)/(3×3)	(256, 1)/(1×1)	-
Scale-3	(512, 512)/(3×3)	(512, 256)/(1×1)	(256, 256)/(3×3)	(256, 1)/(1×1)	-
Scale-4	(768, 768)/(3×3)	(768, 512)/(3×3)	(512, 256)/(1×1)	(256, 256)/(3×3)	(256, 1)/(1×1)

**Table 2:** Performance of the proposed MGCC and other 10 state-of-the-art methods on 4 popular datasets. **Red**, **green** and **blue** indicate the best, the second best and the third best performances. “–” represents no reported.

Datasets	ECSSD [37]					HKU-IS [27]					PASCAL-S [38]					DUT-OMRON [39]				
Methods	Max$F_{\beta }$↑	MAE↓	$F_{\beta }^{w}$↑	$S_{\alpha }$↑	$E_{m}$↑	Max$F_{\beta }$↑	MAE↓	$F_{\beta }^{w}$↑	$S_{\alpha }$↑	$E_{m}$↑	Max$F_{\beta }$↑	MAE↓	$F_{\beta }^{w}$↑	$S_{\alpha }$↑	$E_{m}$↑	Max$F_{\beta }$↑	MAE↓	$F_{\beta }^{w}$↑	$S_{\alpha }$↑	$E_{m}$↑
DRFI [24]	0.782	0.170	0.462	0.720	0.763	0.777	0.145	0.528	0.727	0.832	0.694	0.201	0.469	0.648	0.745	0.664	0.150	0.326	0.697	0.793
RBD [54]	0.716	0.171	0.430	0.695	0.697	0.723	0.142	0.488	0.683	0.736	0.659	0.197	0.429	0.617	0.670	0.630	0.144	0.397	0.668	0.721
MB+ [56]	0.739	0.171	0.428	0.607	0.691	0.728	0.150	0.491	0.534	0.643	0.680	0.193	0.453	0.714	0.814	0.624	0.168	0.386	0.579	0.693
MST [55]	0.731	0.149	0.445	0.601	0.686	0.722	0.168	0.485	0.693	0.753	0.670	0.187	0.458	0.636	0.715	0.600	0.149	0.313	0.653	0.688
SMD [40]	0.760	0.173	0.453	0.716	0.745	0.743	0.156	0.502	0.702	0.796	0.690	0.201	0.463	0.645	0.737	0.624	0.166	0.385	0.686	0.716
LEGS [28]	0.827	0.118	0.805	0.786	0.872	0.767	0.192	0.736	0.743	**0.931**	0.759	0.155	–	0.728	–	0.670	0.204	0.631	0.713	–
MCDL [16]	0.837	0.110	**0.816**	0.803	0.889	0.808	0.091	0.768	0.786	**0.927**	0.743	0.146	**0.787**	0.721	0.706	0.702	**0.088**	**0.670**	**0.752**	0.670
DCL [17]	**0.887**	**0.072**	**0.838**	**0.868**	**0.916**	**0.880**	**0.058**	**0.841**	**0.877**	**0.931**	**0.808**	**0.110**	**0.733**	**0.785**	**0.849**	**0.717**	0.094	0.639	**0.771**	**0.826**
MDF [27]	0.834	0.105	0.810	0.776	0.886	0.814	0.112	0.754	0.810	0.872	**0.768**	0.150	0.704	0.696	0.794	0.694	**0.092**	**0.643**	0.721	**0.820**
ELD [53]	**0.866**	**0.079**	0.786	**0.838**	**0.910**	**0.839**	**0.073**	**0.780**	**0.821**	0.910	**0.771**	**0.126**	0.669	**0.761**	**0.818**	**0.700**	**0.092**	0.596	0.751	0.797
Scale-1	0.783	0.165	0.744	0.732	0.765	0.782	0.143	0.673	0.729	0.834	0.693	0.184	0.682	0.707	0.752	0.672	0.157	0.494	0.701	0.762
Scale-2	0.807	0.143	0.766	0.763	0.803	0.807	0.124	0.715	0.752	0.858	0.734	0.153	0.704	0.725	0.762	0.688	0.135	0.558	0.713	0.794
Scale-3	0.823	0.128	0.783	0.791	0.824	0.825	0.106	0.753	0.790	0.892	0.757	0.138	0.718	0.743	0.778	0.693	0.117	0.583	0.721	0.807
Scale-4	0.835	0.104	0.811	0.803	0.865	0.834	0.091	0.776	0.819	0.913	0.765	0.127	0.730	0.754	0.794	0.699	0.105	0.623	0.739	0.815
**MGCC**(Proposed)	**0.891**	**0.066**	**0.847**	**0.887**	**0.931**	**0.878**	**0.057**	**0.838**	**0.886**	**0.955**	**0.808**	**0.100**	**0.796**	**0.793**	**0.857**	**0.726**	**0.074**	**0.698**	**0.793**	**0.838**
